# Supplementary material for: A Review and Perspective of eDNA Application to Eutrophication and HAB Control in Freshwater and Marine Ecosystems
Source: Microorganisms. 2020 Mar 16;8(3):417. doi: 10.3390/microorganisms8030417 (PMC7143994; doi:10.3390/microorganisms8030417)
Supplement: Supplementary file 1 [file microorganisms-08-00417-s001.zip › microorganisms-730927-SI/Table S1.pdf]

A review and perspective of eDNA application to eutrophication and HAB control in freshwater and marine ecosystems

Qi Liu[1], Yun Zhang[1], Han Wu[1], Fengwen Liu[1], Wei Peng[1], Xiaonan Zhang[1], Fengqin Chang[1], Ping Xie[1, 2] and Hucai Zhang[1, *]

Authors Affiliations: [1] Institute for Ecological Research and Pollution Control of Plateau Lakes, School of Ecology and Environmental Science, Yunnan University, Kunming 650504, China. [2] Donghu Experimental Station of Lake Ecosystems, State Key Laboratory of Freshwater Ecology and Biotechnology, Institute of Hydrobiology, CAS, Wuhan 430072, China

***Corresponding author**: zhanghc@ynu.edu.cn.

Table S1. Representative studies of eDNA application in aquatic ecosystems.

eDNA sample type	PCR amplification region (primers)	Major findings	Citation
Seawater samples	18S V9 (1391_F/1560_R)	Detected aquaculture pathogens from the complex mixture of organisms.	[1]
Water samples from lakes	Specific primers targeting the mitochondrial COI gene	Quantified relative fish abundance using specific primers in twelve lakes.	[2]
Water samples from a freshwater reservoir	18S V4 (SSU_F04/SSU_R22)	Monitored the spatial and temporal dynamics of eukaryotic planktonic species using DNA metabarcoding in a freshwater reservoir.	[3]
Seawater samples	16S V6 (A-967F/B-1046R)	First established the massively parallel V6 tag sequencing strategy to develop a global and in-depth description of the diversity of microbes and their relative abundance in the sea.	[4]
Seawater samples	V4 SSU rRNA (TAReuk454FWD1/TAReukREV3) and V9 SSU rRNA (1391F/EukB)	Detected eukaryotic microbes and rare genotypes in samples from anoxic Norwegian fjord water using two SSU rDNA markers (V4 and V9 region). Both markers identified a wide range of taxonomic groups and the diversity in this region was dominated by dinoflagellates.	[5]
Water samples	Species-specific primers targeting the	Successfully monitored the presence of three invasive crayfishes, *Procambarus*	[6]

Table S1. Representative studies of eDNA application in aquatic ecosystems.

from ponds	mitochondrial COI gene	*clarkii*, *Orconectes limosus* and *Pacifastacus leniusculus*, using eDNA in the ponds of the Brenne Regional Natural Park.	
Water samples from rivers	18S rRNA (1380F/1510R) and 16S rRNA (341F/518R)	Determined the species distribution from the Yangtze River Delta (YRD) through eDNA metabarcoding and identified the major taxonomic lineages as Chlorophyta, Ciliophora and Proteobacteria at family level. PCA indicated that nutrients, such as NO_3^-, NH_4^+, TN and TP, are the main factors determining the community structure of rivers. These data could be used to predict the pollution status based on the OTUs and MLRs.	[7]
Water samples from streams	18S rDNA (D602F/D753R)	Detected the distribution and genetic diversity of the freshwater diatom *Didymosphenia geminata* by combining eDNA and visual assessment in eastern North America and determined that *D. geminata* is an invasive species in the Mid-Atlantic region and is still in an early invasion stage.	[8]
Sediments and water samples from a lake	16S rRNA V4 (515F/B-806R)	Indicated that the bacterial biodiversity is positively correlated with NH_4^+-N in the water and negatively correlated with NO_X-N in the sediment.	[9]
Sediment samples	18S rRNA v9 (1380F/1510R)	Demonstrated that *in situ* eukaryotic communities could be used to reveal	[10]

Table S1. Representative studies of eDNA application in aquatic ecosystems.

		chemical pollution from different land-use types, such as agricultural regions and industrial regions, in sediments from Nanfei River in Anhui Province, China.	
Water samples from a lake	SSU rRNA V1-V3 for *Eukarya* (7F/591R) and for *Bacteria* (FD1/529R)	Analyzed the changes in eukaryotic and bacterial communities and correlations with environmental conditions in Lake Velence. Identified the positive correlation between eukaryotic diversity and DOC or DIC. Showed that the *Microcystis* abundance has a negative correlation with bacterial diversity such as *Actinobacteria*.	[11]
Seawater samples	Elasmobranch specific COI primer set	Detected shark diversity from seawater samples in Atlantic and Pacific tropical ecosystems through eDNA metabarcoding with the specific primers for mitochondrial COI region for the first time.	[12]
Water samples from a coastal ecosystem	18S rRNA V9	Detected the general ecological characteristics of aquatic biodiversity in the coastal ecosystem of Gwangyang Bay and indicated that eDNA could be used to investigate the spatial characteristics of species communities in coastal ecosystems.	[13]
Seawater samples	16S rDNA (16s_Metazoa_fwd/16s_Metazoa_rev)	Analyzed the changes in community composition, diversity and life-history composition linked to the human alteration of upland urbanization and revealed	[14]

Table S1. Representative studies of eDNA application in aquatic ecosystems.

		the interactions between human effects and environmental changes in nearshore environments.	
Water samples from rivers	Standard COI primers	Identified 296 families of eukaryotes and some terrestrial species in river water samples by eDNA metabarcoding in Switzerland and indicated that eDNA in river water could incorporate species biodiversity information from terrestrial and aquatic ecosystems.	[15]
Sediment samples	18S rRNA V7 (960F/NSR1438)	Investigated the temporal rearrangements of and diversity changes in microbial communities with two main stressors: local and global environmental fluctuations.	[16]
Water samples from ponds	Species-specific primers targeting the mitochondrial COI gene	Compared the eDNA methods and traditional methods for detecting crayfish in ponds and suggested that the integration of two approaches could be a better method for the detection of invasive species in waterbodies.	[17]

Reference

1. Peters, L.; Spatharis, S.; Dario, M.A.; Dwyer, T.; Roca, I.J.T.; Kintner, A.; Kanstad-Hanssen, O.; Llewellyn, M.S.; Praebel, K. Environmental DNA: A New Low-Cost Monitoring Tool for Pathogens in Salmonid Aquaculture. *Front Microbiol* **2018**, *9*, doi:Artn 3009, 10.3389/Fmicb.2018.03009.

2. Lacoursiere-Roussel, A.; Cote, G.; Leclerc, V.; Bernatchez, L. Quantifying relative fish abundance with eDNA: a promising tool for fisheries management. *J Appl Ecol* **2016**, *53*, 1148-1157, doi:10.1111/1365-2664.12598.

3. Banerji, A.; Bagley, M.; Elk, M.; Pilgrim, E.; Martinson, J.; Domingo, J.S. Spatial and temporal dynamics of a freshwater eukaryotic plankton community revealed via 18S rRNA gene metabarcoding. *Hydrobiologia* **2018**, *818*, 71-86, doi:10.1007/s10750-018-3593-0.

4. Sogin, M.L.; Morrison, H.G.; Huber, J.A.; Mark Welch, D.; Huse, S.M.; Neal, P.R.; Arrieta, J.M.; Herndl, G.J. Microbial diversity in the deep sea and the underexplored "rare biosphere". *P Natl Acad Sci USA* **2006**, *103*, 12115-12120, doi:10.1073/pnas.0605127103.

5. Stoeck, T.; Bass, D.; Nebel, M.; Christen, R.; Jones, M.D.M.; Breiner, H.W.; Richards, T.A. Multiple marker parallel tag environmental DNA sequencing reveals a highly complex eukaryotic community in marine anoxic water. *Mol Ecol* **2010**, *19*, 21-31, doi:10.1111/j.1365-294X.2009.04480.x.

6. Mauvisseau, Q.; Coignet, A.; Delaunay, C.; Pinet, F.; Bouchon, D.; Souty-Grosset, C. Environmental DNA as an efficient tool for detecting invasive crayfishes in freshwater ponds. *Hydrobiologia* **2018**, *805*, 163-175, doi:10.1007/s10750-017-3288-y.

7. Li, F.L.; Peng, Y.; Fang, W.D.; Altermatt, F.; Xie, Y.W.; Yang, J.H.; Zhang, X.W. Application of Environmental DNA Metabarcoding for Predicting Anthropogenic Pollution in Rivers. *Environ Sci Technol* **2018**, *52*, 11708-11719, doi:10.1021/acs.est.8b03869.

8. Keller, S.R.; Hilderbrand, R.H.; Shank, M.K.; Potapova, M. Environmental DNA genetic monitoring of the nuisance freshwater diatom, Didymosphenia geminata, in eastern North American streams. *Divers Distrib* **2017**, *23*, 381-393, doi:10.1111/ddi.12536.

9. Wan, Y.; Ruan, X.H.; Zhang, Y.P.; Li, R.F. Illumina sequencing-based analysis of sediment bacteria community in different trophic status freshwater lakes. *Microbiologyopen* **2017**, *6*, doi:ARTN e450, 10.1002/mbo3.450.

10. Xie, Y.W.; Wang, J.Z.; Yang, J.H.; Giesy, J.P.; Yu, H.X.; Zhang, X.W. Environmental DNA metabarcoding reveals primary chemical contaminants in freshwater sediments from different land-use types. *Chemosphere* **2017**, *172*, 201-209, doi:10.1016/j.chemosphere.2016.12.117.

11. Bell, T.A.S.; Sen-Kilic, E.; Felfoldi, T.; Vasas, G.; Fields, M.W.; Peyton, B.M. Microbial community changes during a toxic cyanobacterial bloom in an alkaline Hungarian lake. *Anton Leeuw Int J G* **2018**, *111*, 2425-2440, doi:10.1007/s10482-018-1132-7.

12. Bakker, J.; Wangensteen, O.S.; Chapman, D.D.; Boussarie, G.; Buddo, D.; Guttridge, T.L.; Hertler, H.; Mouillot, D.; Vigliola, L.; Mariani, S. Environmental DNA reveals tropical shark diversity in contrasting levels of anthropogenic impact. *Sci Rep-Uk* **2017**, *7*, doi:Artn 16886, 10.1038/S41598-017-17150-2.

13. Jo, H.; Kim, D.-K.; Park, K.; Kwak, I.-S. Discrimination of Spatial Distribution of Aquatic Organisms in a Coastal Ecosystem Using eDNA. *Applied Sciences* **2019**, *9*, 3450.

14. Kelly, R.P.; O'Donnell, J.L.; Lowell, N.C.; Shelton, A.O.; Samhouri, J.F.; Hennessey, S.M.; Feist, B.E.; Williams, G.D. Genetic signatures of ecological diversity along an urbanization gradient. *Peerj* **2016**, *4*, doi:Artn E2444, 10.7717/Peerj.2444.

15. Deiner, K.; Fronhofer, E.A.; Machler, E.; Walser, J.C.; Altermatt, F. Environmental DNA reveals that rivers are conveyer belts of biodiversity information. *Nat Commun* **2016**, *7*, 1-9, doi:Artn 12544, 10.1038/Ncomms12544.

16. Capo, E.; Debroas, D.; Arnaud, F.; Guillemot, T.; Bichet, V.; Millet, L.; Gauthier, E.; Massa, C.; Develle, A.L.; Pignol, C., et al. Long-term dynamics in microbial eukaryotes communities: a palaeolimnological view based on sedimentary DNA. *Mol Ecol* **2016**, *25*, 5925-5943, doi:10.1111/mec.13893.

17. Treguier, A.; Paillisson, J.M.; Dejean, T.; Valentini, A.; Schlaepfer, M.A.; Roussel, J.M. Environmental DNA surveillance for invertebrate species: advantages and technical limitations to detect invasive crayfish Procambarus clarkii in freshwater ponds. *J Appl Ecol* **2014**, *51*, 871-879, doi:10.1111/1365-2664.12262.
